# Optimisation of Alginate Extraction and Characterisation of Polysaccharides from Brown Seaweed from the Portuguese Coast

**DOI:** 10.3390/md24020060

**Published:** 2026-02-01

**Authors:** Joana Corrêa Mendes, Joana F. A. Valente, Fani Sousa, Raul Bernardino, Susana Bernardino, Clélia Afonso, Bárbara Chagas

**Affiliations:** 1 ESTM—School of Tourism and Maritime Technology, Polytechnic of Leiria, Rua do Conhecimento 4, 2520-614 Peniche, Portugal; joana.m.fernandes@ipleiria.pt (J.C.M.); raul.bernardino@ipleiria.pt (R.B.); susana.bernardino@ipleiria.pt (S.B.); 2 CDRSP-PL—Centre for Rapid and Sustainable Product Development, Polytechnic of Leiria, 2411-901 Leiria, Portugal; joana.valente@ipleiria.pt; 3 RISE-Health, UBI—Department of Medical Sciences, Faculty of Health Sciences, University of Beira Interior, Av. Infante D. Henrique, 6200-506 Covilhã, Portugal; fani.sousa@fcsaude.ubi.pt; 4 MARE—Marine and Environmental Sciences Centre/ARNET–Aquatic Research Network, School of Tourism and Maritime Technology, Polytechnic of Leiria, Edifício Cetemares, Av. Porto de Pesca, 2520-641 Peniche, Portugal; 5 LSRE-LCM—Laboratory of Separation and Reaction Engineering-Laboratory of Catalysis and Materials, School of Tourism and Maritime Technology, Polytechnic Institute of Leiria, 2520-614 Peniche, Portugal; 6 ALiCE—Associate Laboratory in Chemical Engineering, Faculty of Engineering, University of Porto, Rua Dr. Roberto Frias, 4200-465 Porto, Portugal

**Keywords:** brown seaweeds, alginate, polysaccharides, extraction methods, *Saccorhiza polyschides*, *Sargassum muticum*

## Abstract

Alginate is a widely used and versatile biopolymer with an ever-expanding range of applications in the pharmaceutical and biomedical industries. This highlights the importance of developing sustainable and renewable production sources. Conventional extraction methods, although effective, are often energy-intensive and rely on harsh chemicals. In this context, brown algae are a promising alternative due to their abundance and renewability. This study investigated the potential of *Saccorhiza polyschides* and *Sargassum muticum* as sources of sodium alginate (SA), thus optimising an extraction process that combines acid treatment with an alkaline step. The extracted biopolymers were characterised using FTIR, H-NMR, STA, SEM/EDX, viscosity measurements, dynamic light scattering, and spectrophotometric assays of residual polyphenols and proteins. The optimised extraction conditions produced yields above 20% of high-purity alginate. When compared with commercial SA, the extracted materials showed comparable quality while relying on a simplified, solvent-reduced protocol that improves process efficiency and reduces the environmental impact. These results demonstrate that *S. polyschides* and *S. muticum* are promising, locally available sources of high-quality sodium alginate, and that industrially relevant yields (>20%) can be achieved through an environmentally conscious two-step extraction process.

## 1. Introduction

Alginates are a family of hydrophilic or anionic polysaccharides derived from brown seaweed (Phaeophyceae). They are of significant interest due to their wide range of applications in various industries, including food, pharmaceuticals, textiles, and biotechnology [1]. The main polysaccharides found in the cell walls of brown algae are alginate (accounting for 40% of seaweed’s dry matter), fucoidan, and laminarin [2]. These polysaccharides provide a flexible mechanical structure that protects seaweed from damage caused by strong currents [3]. These biopolymers are primarily composed of randomly distributed (1 → 4) linked β-D-mannuronic acid (M) and α-L-guluronic acid (G) residues that compose carbohydrate chains. These uronic acids can form homogeneous blockchains of MM (M blocks) or GG units (G blocks), as well as chains with alternate blocks of mannuronic acid and guluronic acid (MG blocks) [4]. The structure of alginate, which is mainly defined by the sequence pattern, G-block length, M/G ratio, and molecular weight, is key in determining its physicochemical and technological properties [5], such as gel formation, viscosity modification, and bioadhesion, which also influence the biological activity [6]. These structure–function relationships demonstrate why alginates are increasingly being investigated not only as industrial biopolymers but also as functional biomaterials with biomedical relevance.

The versatility of alginates in producing hydrogels is crucial to their wide range of applications. Alginates quickly form crosslinks in the presence of divalent ions, such as Ca^2+^, due to interionic interactions with G residues. This is one of the most common methods of producing hydrogels, alongside other techniques such as covalent crosslinking, thermal gelation, and cell crosslinking. For decades, these crosslinking methods have been used to encapsulate biomolecules and cells. Due to the biocompatibility, low cost, and reduced toxicity of ionic gelation mechanisms, alginates are often chosen for medical applications, including drug delivery, wound dressings, and tissue engineering [7,8,9]. Nevertheless, the performance of alginate-based hydrogels depends heavily on the source and extraction method, as these directly affect the composition and physicochemical properties.

*Saccorhiza polyschides* is a large brown seaweed that is commonly found along European coastlines, particularly on subtidal rocky reefs at the lowest shore. It is particularly prevalent in the coastal waters of northwest Portugal. This seaweed has an annual life cycle, with sporophytes growing rapidly in spring and summer before experiencing strong decay in autumn and being detached by winter storms [10,11,12]. *S. muticum* is a fast grower, with very efficient photosynthetic activity and a high reproductive capacity. Its effective sexual reproduction contributes to its efficient dispersion [13,14]. These different characteristics provide a clear understanding of its evolutionary and establishment success. *S. muticum* is a major invasive macroalga that significantly disrupts ecosystem services. It heavily impacts coastal and recreational fisheries by tangling fishing lines, nets, and gear. *S. muticum* is rich in carbohydrates and proteins and has notable antioxidant properties [15]. The morphology, composition, and distribution of the species can affect the yield and quality of the extracted alginate. It has also been verified that the time and area of collection can impact the yield of alginate for the same species.

The contrasting ecological profiles of *S. polyschides* (native and abundant species) and *S. muticum* (an invasive and disruptive species) make them highly relevant case studies for sustainable alginate extraction, demonstrating how local resources can be valued while promoting the biotechnological use of invasive biomass. These two ecologically contrasting brown algae, a native opportunistic species (*S. polyschides*) and an invasive species (*S. muticum*), were selected as abundant, underutilised biomass sources to assess their potential as sustainable feedstocks for alginate production [16,17].

Traditionally, the extraction of alginates involves the use of chemical treatments with sodium carbonate and calcium chloride, followed by acid precipitation [18]. While effective, these conventional methods raise several environmental and economic concerns. The extensive use of chemicals, high energy consumption, and generation of waste effluents contribute to environmental degradation and increase the overall carbon footprint of the production process. In response to these challenges, this research work has developed an alternative extraction method that is more sustainable and environmentally friendly. This optimised method proposes an optimised extraction protocol applied to two ecologically distinct brown algae species. It has the dual goal of maximising the alginate yield and characterising its physicochemical properties under more sustainable conditions as well as guaranteeing the preservation of the marine environment.

This study focuses on the valorisation of the invasive macroalga *Sargassum muticum* and the opportunistic species *Saccharina polyschides*, both widely distributed along the Portuguese coast, as promising and sustainable sources of high-purity, high-quality alginate. We propose that this biopolymer can be efficiently extracted using a simple, rapid, and environmentally friendly approach, avoiding hazardous solvents and sophisticated technologies, while still achieving high extraction yields (>20%). Beyond the development of a more sustainable extraction protocol tailored to two ecologically distinct brown algae, this work provides a comprehensive comparative assessment of the obtained alginates, encompassing the extraction yield, purity, structural characteristics, and functional properties. This integrated evaluation aims to highlight the potential of these species as alternative, environmentally responsible sources of alginate for future biopolymer applications.

## 2. Results and Discussion

The use of biomaterials such as alginate holds significant promise in several biomedical applications due to its biocompatibility, non-toxicity, and versatile properties. The recovery of these biomaterials from sustainable sources, such as invasive algae, not only provides an eco-friendly and abundant supply of raw materials but also helps mitigate the environmental impact of invasive species. The processes of extraction, purification, and characterisation of alginate can directly influence its quality and, consequently, its applicability in fields such as tissue engineering, drug delivery, and wound healing. Thorough physicochemical characterisation is crucial to ensure that the alginate meets the required standards for specific applications, thereby maximising its efficacy and safety. This work focused on the development of an optimised alginate extraction protocol using the brown seaweed *S. polyschides* and *S. muticum* as sources. The physical and chemical characteristics of the biopolymer, including functional groups, thermal behaviour, glucuronic and mannuronic acid composition, and surface morphology, were also analysed. The primary goal was to ensure the efficiency of the extraction methodology while maintaining product quality and process sustainability, emphasising the use of these algae to obtain a high-quality product.

### 2.1. Alginate Extraction Optimisation

The efficiency of the extraction process is influenced by several parameters, including the temperature, alkaline concentration, time, pH, and their interplay with the characteristics of the species. Acid treatment eliminates non-targeted compounds present in seaweed, such as polyphenols and easily degradable polysaccharides. Moreover, the time required for the extraction process not only impacts the overall yield but also affects the rheological properties of alginate. Overall, when the time of extraction increases, the contact between the cells of the macroalgae and the solvent is improved, allowing for the disruption of the cells in a more efficient way [15]. The water-insoluble alginic acid present in the algae cell wall is converted into water-soluble salts and then precipitated, purified, and dried [2].

**Figure 1:**
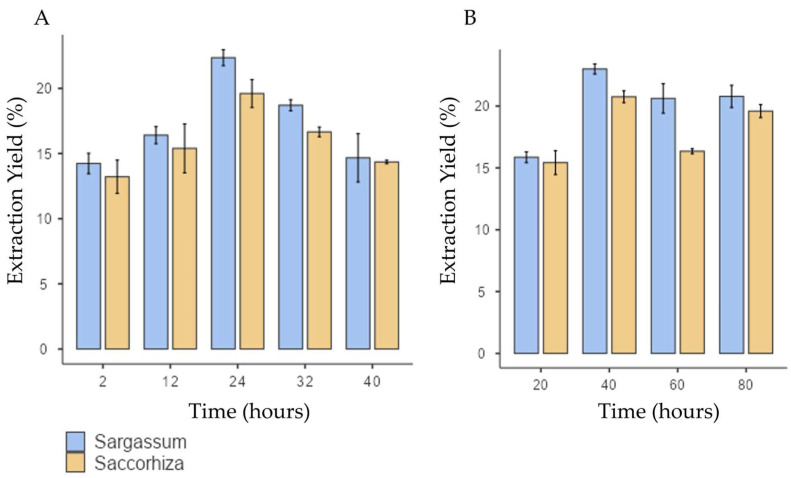
Extraction yield for *S. muticum* and *S. polyschides* for each variable under test: (**A**) time (hours) for acid treatment; (**B**) temperature of alkaline extraction. Results are expressed in means ± SD (*n* = 4).

Concerning the extraction yield, both *S. polyschides* and *S. muticum* species exhibited a similar response to variable extraction time. The extraction yield increased with longer extraction periods, with maximum performance achieved at 24 h, and with values of 21% ± 1.01 and 23.2% ± 1.23 for *S. polyschides* and *S. muticum*, respectively (as seen in Figure 1). This demonstrates that prolonged extraction enhances the yield up to an optimal point, but exceeding this threshold leads to yield decline, likely due to polymer depolymerisation.

However, if the extraction time is extended, a decline in yield is observed for both species. Notably, the standard deviation in the *S. muticum* showed poor reproducibility, possibly due to the depolymerisation of the polymer chain, rendering it less prone to precipitation.

In the literature, the predictable SA yield of *S. polyschides* spans from 9% to 20% [19,20]. In similar conditions to those of this study, Gomes and collaborators [20] reported a yield of 11% for this same species. This optimisation enabled *S. polyschides* to reach alginate concentrations like those of the main alginophytes used in industry worldwide (20–41% dw), despite it not typically being used for this purpose [21]. Kaidi et a,. [19] reached a yield of SA of 20% using formalin as pretreatment, in a protocol extended for 72 h. Also, similar yields were obtained by Gomes et al., [20] using microwave-assisted extraction. In the present study, the optimised protocol yielded 21% of alginate with no additional pretreatments, downstream steps, or assisted extraction technologies. This highlights a key advantage of the present work: achieving industrially relevant yields (≈21%) without the need for harsh solvents or energy-intensive methods, reinforcing the protocol’s eco-friendly character. This allowed for a decrease in time, energy, and chemical consumption, rendering the process fast, simple, and effective.

Compared to other species in the Sargassum genus, *S. muticum* is known for having one of the lowest percentages of alginate in its composition. Reported values range from 6.8% [22] to 18% [19]. In a similar and optimised procedure, Mazumber et al. achieved a 13% extraction yield by incorporating a pretreatment step with formaldehyde. The use of strong solvents, such as chloroform, formaldehyde, or petroleum ether, or technologies like microwave-assisted or enzyme-assisted extraction, can result in higher extraction yields. However, these methods are often more time-consuming and costly and can be less environmentally friendly than using green solvents.

The source profoundly influences the extraction yield, as various algae exhibit distinct contents of alginate, viscosity, molecular weight, and varying M/G ratios. It is important to emphasise that the values obtained in this experiment are a culmination of these diverse variables, compounded with all of the process-related factors like the algae species, seasonality, geographic distribution of the species, extraction method, purification method, among many others.

The temperature used in alkaline extraction is a critical factor, typically falling within the range of 20 °C to 100 °C. However, in this study, temperatures exceeding 80 °C were not explored, since optimal yields were achieved below this threshold. Increasing the extraction temperature can enhance the release of alginate from the brown algae matrix. Nonetheless, it is important to note that higher temperatures can also accelerate the degradation of alginate polymer chains, thereby resulting in the reduced viscosity of the alginate solution [18,23].

In this study, the extraction yield reached its maximum at 40 °C for both algae species, as seen in Figure 1B. For higher temperatures, there was a slight decrease in the extraction yield from *S. polyschides* and a much more pronounced decline for *S. muticum*, indicating a higher susceptibility of this species to temperature. This observation suggests that temperatures exceeding 40 °C push the degradation of polymer chains beyond the optimal point, leading to reduced precipitation and, consequently, to a lower yield of SA. Notably, both species exhibited a similar response to the temperature stimulus, highlighting the consistency of their behaviour in the extraction process. These findings underscore that moderate temperatures (≈40 °C) are sufficient to optimise the yield, while higher values negatively impact polymer integrity, particularly in *S. muticum*. This indicates species-specific sensitivity and aligns with previous reports on alginate thermal degradation.

In the literature, Mazumder and coworkers found the optimal temperature for alginate extraction to be 86 °C for *S. muticum*, yielding an extraction rate of 13%. On the other hand, for *S. polyschides*, temperatures of 40 °C are also described as optimal but with a lower extraction yield (approximately 11% in the literature) than that reported in this study. An additional advantage of the procedure described here is the fact that no pretreatment with chloroform or other harsh chemicals was applied. Considering that this optimised protocol follows a traditional method without requiring additional steps or assisted extraction technologies, it can be concluded that this approach enabled a simple yet effective extraction of alginate from *S. polyschides* and *S. muticum*.

**Figure 2:**
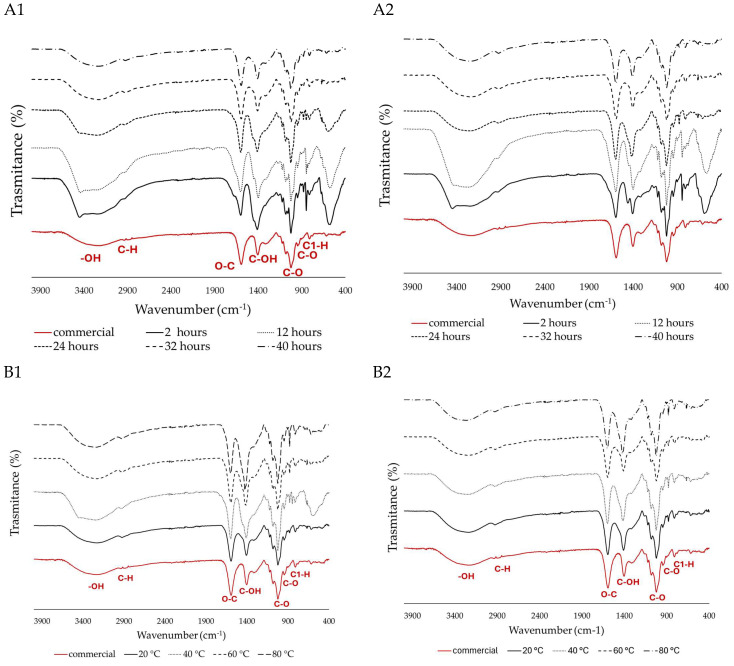
FITR spectra of extracted SA, and acid treatment optimisation for the time points 2, 12, 24, 36, and 40 h from (**A1**) *S. polyschides* and (**A2**) *S. muticum*. Alkaline extraction optimisation for temperatures of 20, 40, 60, and 80 °C from (**B1**) *S. polyschides* and (**B2**) *S. muticum*. The different lines represent the spectra obtained under each experimental condition, and the red line corresponds to the commercial SA used as the control.

For the statistical analysis, the outliers were removed and Levene’s test confirmed variance homogeneity (*p* > 0.05). Two-way ANOVA showed that time, temperature, and algae species significantly affect the extraction yield, with a notable interaction between algae type and temperature (Table 1 and Table 2). While the algae×time interaction was not significant, the algae×temperature interaction revealed that extraction performance depends on both species and environmental conditions. These findings highlight the importance of both individual variables and their interplay, aligning with observable differences in algae behaviour under varying temperatures (Figure 2).

### 2.2. Physicochemical Characterisation

#### 2.2.1. Polyphenol Quantification

Polyphenols play a key role in the chemical defence against herbivores in brown seaweed. Since they are naturally present in algae, it is expected that this kind of contaminant could be found in the extract. It is well established that purity is a crucial quality attribute for SA, particularly in biomedical applications, as it can be associated with higher biocompatibility, consistency, safety, functionality, and regulatory compliance, ultimately contributing to the success and reliability of biomedical products and therapies. To this end, the World Health Organisation reports that polyphenol can be dangerous for humans, so it is of great importance to remove these compounds from alginates before their implantation [24]. Normally, phenolic compounds are removed from the cell matrix during acid treatment; however, measuring this parameter can be a helpful tool to evaluate the efficiency of the extraction process. Commonly, the polyphenol content is expressed as the gallic acid equivalent (GAE) and calculated using a standard curve. In this study, all purification procedures were found to significantly decrease the polyphenol content in the alginate (Table 3), even without the use of harsh chemicals like formol/chloroform. The value reported until now using SA extracted from *S. polyschides* could reach 66.7 mg (GAE)/g [21]. In the present study, upon purification, the values obtained were 0.08 mg (GAE)/g, being very close to the value obtained for commercial alginate, reflecting the efficiency of the process in obtaining a pure SA sample. The drastic reduction in polyphenols to levels comparable with commercial alginate (≈0.07 mg GAE/g) highlights the efficiency of the protocol in producing high-purity SA that is suitable for biomedical use, even in the absence of harsh chemicals like chloroform. In the literature, the values of phenolic content for *S. muticum* range from 71 mg (GAE)/g extract to 0.236 mg (GAE)/mL [25,26,27]. *S. muticum* is an excellent source of polyphenols, like gallic [28], protocatechuic, and gentisic acids [29], which can explain the higher phenolic content of this sample when compared with *S. polyschides* or with the commercial option.

#### 2.2.2. Total Protein

Depending on the brown seaweed source, the distribution of macrocomponents of algae can be up to 50% carbohydrates (alginate, carrageenan, and agar) and up to 40% proteins [26]. Due to the similar amounts of these two coexisting components, the contamination of the bulk alginate by proteins is not surprising. The presence of certain proteins in biomedical devices is known to elicit a strong host immune reaction, making their removal from the alginate before implantation crucial. Commercial formulations of SA often exhibit vestigial traces of protein, as it is considered an impurity related to a poorer extraction and/or purification process. However, protein impurities may also influence the mechanical properties, gelation behaviour, or drug release kinetics of alginate-based formulations [27].

The protein content in *S. muticum*, like other seaweeds, can vary depending on factors such as the season, geographic location, seaweed age, and environmental conditions. However, studies have reported that *S. muticum* generally has a protein content ranging from approximately 10% to 20% [5,25,30], and that *S. polyschides* can contain protein levels ranging from approximately 6% to 15% [24] of their dry weight. *S. muticum* has the highest protein content, with a value of 0.069% ± 0.02, which is more than four times the value for *S. polyschides* (0.016% ± 0.01) and more than nine times the value for the commercial alginate (0.007% ± 0.01) (Table 4). This suggests that, while *S. muticum* yields a higher alginate content, it also retains more protein contaminants, potentially requiring additional purification. In contrast, *S. polyschides* produced extracts with lower protein levels, indicating greater suitability for biomedical-grade applications. This could also suggest that the extraction and purification process is less effective for this species when compared with *S. polyschides.* The protein content is typically eliminated with acid treatment and temperatures, and the obtained results provide an indication of the efficiency of the extraction of SA with a high purity for both algae species, with a better performance at reducing the levels of polyphenol contaminants.

#### 2.2.3. Molecular Weight and Viscosity

Viscosity and molecular weight are crucial parameters that influence the properties and applications of alginate. They can vary depending on all of the parameters previously described, especially the source of alginate and the extraction process. The literature reports that, for alginates extracted for longer than 2 h, there is a significant decline in their dynamic viscosity, suggesting that an extended extraction period may promote the catalysed depolymerisation of alginate molecules, resulting in a reduction in viscosity [31]. Truus et al., (2001) [32], found that both temperature and time have a detrimental effect on viscosity, and Prokoph and Wang observed a 10–30% decrease in the viscosity after applying the procedure described by Klock and coworkers, with some optimisations [33].

In this study, it was verified that commercially available alginate has a significantly higher viscosity and molecular weight, although the origin is unknown. Typically, cold-water algae present low-viscosity SA [34]. Considering the described phenomenon, we would expect to observe low viscosities for both SA extracts. Furthermore, higher molecular weight SA polymers tend to exhibit higher viscosity. This occurs because longer polymer chains are more prone to entanglement and can form more extensive networks, resulting in increased resistance to flow and, consequently, higher viscosity. The molecular weight of commercially available SA ranges between 32,000 and 700,000 Daltons (=g/mol) [7]. Increasing the molecular weight of alginate can improve the physical properties of the resultant gels. However, an alginate solution formed from a high-molecular-weight polymer becomes more viscous, which is often undesirable, as it creates a brittle gel and makes crosslinking controllable.

The viscosity and molecular weight of the extracted samples were similar and relatively low (Table 5) due to the previously described phenomenon. *S. polyschides* and *S. muticum* have a low viscosity and molecular weight, making them more desirable to applications where the homogeneity of the gel is pivotal. Low viscosity and molecular weight, although below some commercial references, may represent an advantage for specific biomedical applications (e.g., homogeneous gels and controlled drug release), reinforcing the versatility of the extracted alginates. Commercially available alginate has a much higher viscosity and molecular weight, although the origin is unknown.

For therapeutic biomolecules, stability is a key quality attribute that is important for establishing drug-like properties and applicability for use in humans. The diffusion interaction parameter (kD) reveals attractive or repulsive forces, particularly those leading to irreversible aggregation [35]. Positive kD values indicate attraction or association, while negative kD values indicate repulsion or steric hindrance [36]. A kD value close to zero (whether positive or negative) indicates that the hydrodynamic radius (*R*h) is like the radius of gyration (*Rg*), which is related to colloidal stability. This suggests minimal interaction or association between molecules in the solution, meaning they remain relatively stable. This is precisely what was observed in this study [37], where all three types of SA showed similar results, indicating that the polymer molecules do not exhibit particularly strong attractive or repulsive forces in this solution.

#### 2.2.4. FTIR Analysis

FTIR analysis was performed to identify the functional groups of SA, compare the different samples, and identify the typical peaks. Additionally, as a control experiment, commercial SA was used (Figure 2).

The peaks around 3344 and 2925 cm^−1^ are assigned to –OH stretching and a weak aliphatic C–H stretching band, respectively, and are typically associated with water content. The broad bands at 1600–1610 cm^−1^ were suggested to be associated with the O–C–O car-boxylate asymmetric stretching [38]. The bands located at 1400–1428 cm^−1^ were assigned to the C–OH deformation vibration with the involvement of the symmetric stretching vibration of O–C–O. The lack of peaks around 1200–1300 cm^−1^ indicates that there is no stretching vibration of the sulphate (SO_3_) group, which indicates a high purity. The sharp peak observed at 1040 cm^−1^ can be attributed to the elongation of the C–O groups [39]. The anomeric region (950 to 750 cm^−1^) is the most discussed in carbohydrates, where, around 930–950 cm^−1^, the peaks are assigned to the C–O stretching vibration of uronic acid residues, and the ones recorded between 871 and 883 cm^−1^ are attributed to the C1–H deformation vibration of mannuronic acid residues [30,39].

Across several extraction time points, both algae exhibited a comparable response, characterised by the presence of characteristic peaks in their spectra. However, beyond the 12 h mark, a decrease in noise is observed in the anomeric region (below 800 cm^−1^) (Figure 2(A1,B1)). When comparing two or more samples, lower noise indicates better spectral quality and higher purity. In this case, the samples past the 12 h mark of acid treatment become more like the commercially available ones. Additionally, a distinct peak around 1400 cm^−1^, associated with C–OH deformation, was more prominent in *S. polyschides* compared to *S. muticum*. These changes could be due to differences in composition, molecular weight (as a higher peak at 1400 cm^−1^ may signify a broader range of molecular weights), or degree of polymerisation [40,41,42].

Although no significant difference is seen when different temperatures are applied for *S. muticum* (Figure 2(B2)), this alga loses some of the anomeric noise when acid treatment times longer than 24 h are used. *S. polyschides* is seen to lose the unspecific noise upwards of 20 °C and 12 h (Figure 2(A2)).

#### 2.2.5. Simultaneous Thermal Analysis (STA)

To evaluate the thermal stability and overall thermal behaviour of the polymer, STA was performed over a temperature range from 30 °C to 900 °C. By comparing the obtained results with literature reviews and commercially available standards, it was possible to gain insights into the polymer’s purity. The data from the STA were further analysed separately using thermogravimetric analysis (TGA) for thermal degradation and differential scanning calorimetry (DSC) for detailed thermal behaviour.

**Figure 3:**
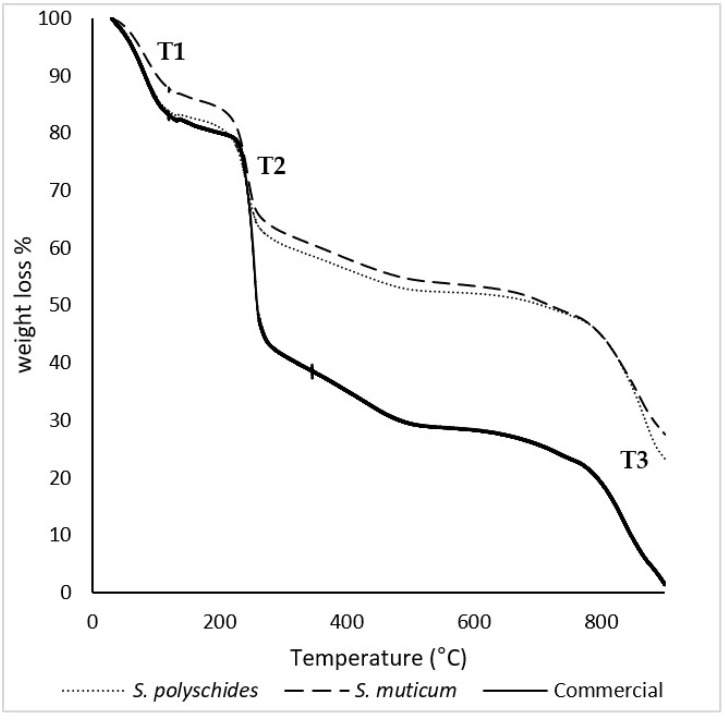
TGA thermograms of *S. polyschides* SA, *S. muticum* SA, and commercial SA. The results are expressed as a percentage of weight loss per temperature. T1, T2, and T3 correspond to the main thermal events observed during the degradation process, namely the initial moisture release (T1), the decomposition of organic constituents (T2), and the final degradation of more thermally stable fractions (T3).

The thermal behaviour of commercial, *S. polyschides*, and *S. muticum* SA was studied through TGA under nitrogen atmosphere, and the relevant degradation temperatures were calculated from the data represented in Figure 3. The TGA curves show the mass loss of the samples throughout their thermal degradation. The results allowed for the identification of three separate thermal events. At the beginning of thermal degradation (T1), a loss of mass was observed, which could be attributed to moisture or coordination water, corresponding to a loss of around 10–15% of the sample’s mass. The second thermal event (T2) happened between 230 °C and 260 °C, with an associated mass loss of 30–40%, which was attributed to the decrosslinking of the polymer networks. The third thermal event (T3) occurred between 760 °C and 900 °C and corresponded to degradation and carbonisation of the remaining residue, leading to the formation of char [43,44].

Table 6 presents the temperatures of initial degradation (T onset) and the peak and maximum temperature degradation (T max) for all thermal events recorded in the thermograms. The T onset, peak, and T max for all the samples were similar, which indicates a high purity of the samples [40,44,45]. SA has a degradation temperature around 210 °C, which corresponds to the onset temperature calculated for the second thermal event, for all samples [41]. According to the literature, these degradation stages could correspond to SA thermal dehydration, followed by the formation and degradation of Na_2_CO_3_, respectively [42].

The temperature at which 50% weight loss occurred was also found to be about 730–770 °C, as described in the literature [39]. Moreover, the glass transition temperature (Tg) is a key performance indicator, as it defines the flexibility and mobility of the chain segmental motion [46]. The Tg and melting temperature (Tm) of the biopolymer electrolyte membrane are obtained by performing DSC analysis; however, SA is an amorphous polymer with an irregular structure and does not crystallise [47].

**Figure 4:**
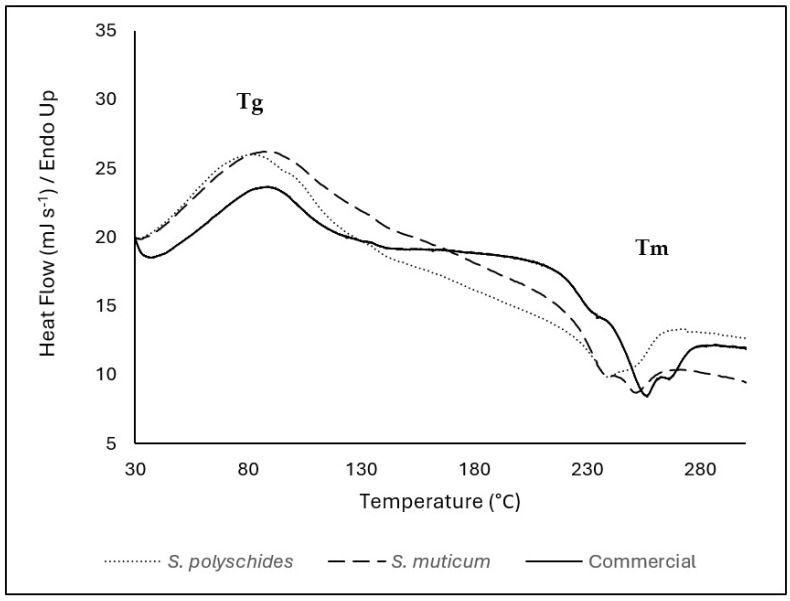
DSC thermogram of SA from *S. polyschides*, *S. muticum*, and commercial SA; results are expressed as heat flow as a function of temperature. The event marks are represented by the temperature of the peak. The event marks are represented by the temperature of the peak. Tg corresponds to the glass transition temperature, and Tm corresponds to the melting temperature identified in the thermogram.

The thermal properties of SA were evaluated considering the temperature range of 30–900 °C; however, since there was no thermal event that occurred above 300 °C, the DSC thermogram was presented in the range between 30 and 300 °C (Figure 4). The Tg of all SA samples were represented by an endothermic peak at around 70–80 °C (69 °C for *S. polyschides*, 75 °C for *S. muticum*, and 76 °C for commercial). The endothermic peaks are correlated with the loss of water associated with the hydrophilic groups of alginates [47]. SA contains hydroxyl and carboxylate groups, which form very strong inter- and intramolecular hydrogen bonding and show a wide transition, as seen by a broader peak presented in Figure 4 [45]. Around 240–250 °C, SA shows a very weak and small melting peak. These exothermic peaks resulted from degradation due to dehydration and depolymerisation reactions, most probably due to the partial decarboxylation of the protonated carboxylic groups, CO_2_ elimination, and chain scission. These peaks are also correlated with the thermal events seen in TGA [38,48,49]. In this study, the differences in peaks between algae can be due to the different compositions, water content, and molecular weight of the extracted SA [50].

In this study, there are slight differences between the onset and maximum temperatures for each thermal event; however, all samples followed the same patterns, showing a similar chemical structure and thermal behaviour. The successful isolation of SA from brown algae is supported by DSC, which shows similar melting temperatures for pure SA and isolated alginate, namely, 243.4 °C, 237 °C, and 249 °C for pure SA, isolated SA from *S. polyschides*, and *S. muticum*, respectively.

#### 2.2.6. SEM and EDX

**Figure 5:**
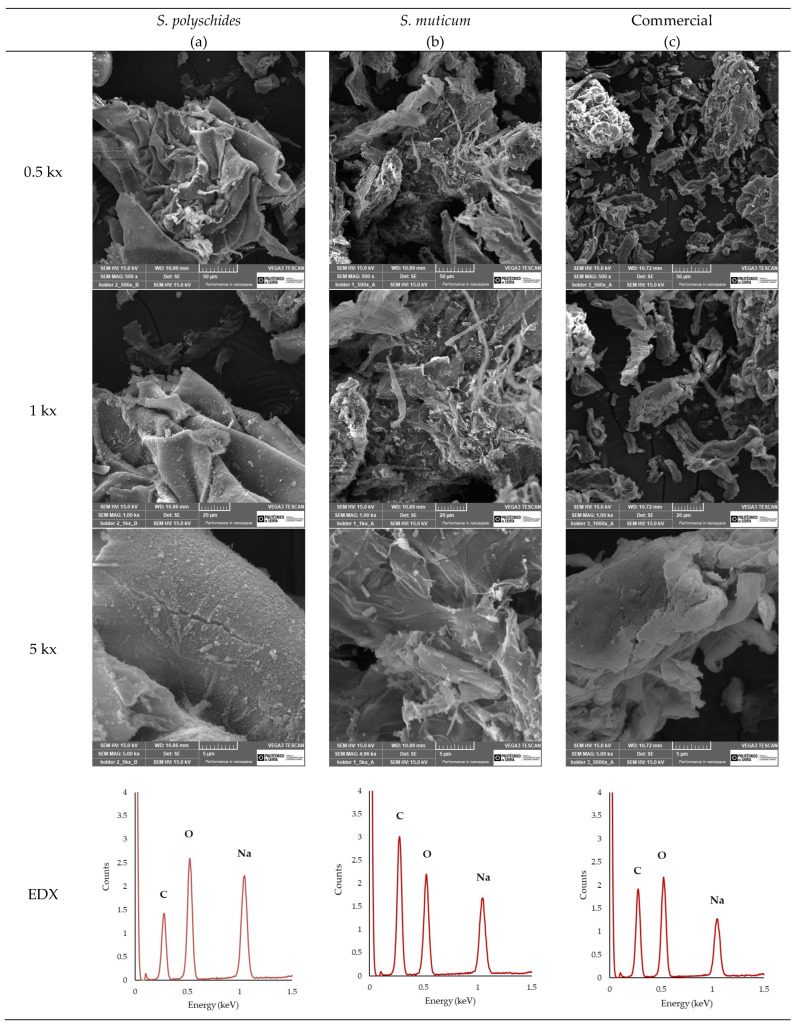
SEM images of (**a**) SA from *S. polyschides*, (**b**) SA from *Sargassum muticum*, and (**c**) commercial SA at different magnifications, and their respective EDX spectra.

SEM analysis (Figure 5) was performed to examine the surface morphology of the extracted SA and compare it with that of the commercially available one. Moreover, EDX analysis enables a comparative analysis of the elemental composition. As seen in Figure 5, the EDX analysis allowed for the identification and quantification of the peaks at 0.28, 0.50, and 1.04 keV, corresponding to carbon, oxygen, and sodium, respectively [51]. The Na/C ratio could be indicative of the influence of the surface appearance of SA, with a higher Na/C ratio showing a smoother surface morphology due to the more extended and less compacted molecular structure. Smoother surfaces, like those observed for *S. polyschides*, indicate that the polymer has a low porosity [52]. A lower Na/C ratio promotes stronger gel formation due to the more efficient crosslinking between alginate chains, leading to denser gel networks. Typically, a higher Na/C ratio results in weaker gel formation compared to lower ratios, leading to a more uniform gel. A weaker gel may be desirable, as it allows for easier manipulation of gel properties, such as the degradation rate or mechanical strength, and tends to have higher solubility due to the increased presence of sodium ions. The higher sodium content contributes to better stability, particularly in storage conditions, where the moisture content or environmental factors may compromise the material’s integrity. However, the ability to form a gel and its properties highly depend on other parameters, such as the M/G ratio, viscosity, molecular weight, and chain conformations [53]. Considering the results, the SA from *S. polyschides* showed a much higher Na/C ratio when compared with the commercial one (0.6255, which was two times higher than the commercial one), as seen in Table 7, thereby aligning with the SEM images acquired.

#### 2.2.7. ^1^H NMR

The structure of alginate is mainly defined by the sequence pattern, G-block length, M/G ratio, and molecular weight, which are key in determining its physicochemical and technological properties [5]. Alginate with a high G content can form brittle and strong gels, while alginate rich in M blocks makes softer and more elastic gels. MG blocks give rise to alginate shrinkage and higher flexibility. In addition, alginate is unable to form gels if the molar fraction of the G blocks is less than 20–25% [54].

^1^H-NMR spectroscopy is the most reliable method for determining the alginate composition and block structure, providing values such as FM, FG, diad frequencies, M/G ratio, and triad frequencies [53,55,56]. The M/G ratio is crucial for predicting alginate’s application, as it influences the gel properties. Gel formation mainly occurs through G block interactions in the presence of divalent cations, with MG blocks forming weaker junctions. Crosslinking can occur via GG/GG, MG/MG, or GG/MG junctions. High M/G ratios yield soft, elastic gels, while low ratios result in strong, rigid gels [57].

**Figure 6:**
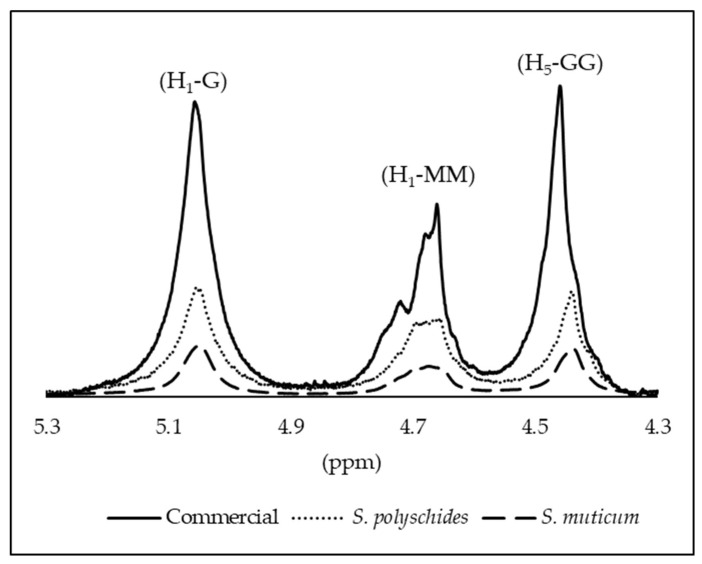
Assignment of the H1 and H5 signals for M and G residues; for all the alginate samples.

The structural characterisation of the samples was performed by ^1^H NMR, and the alginate composition was determined by evaluating the anomeric proton region. The spectra exhibited the characteristic signals of alginates (Figure 6), including the anomeric proton of guluronic acid (G1) at 5.0–5.1 ppm, and the overlapping signals of the mannuronic acid anomeric proton (M1) together with the H-5 proton of G residues (G5) in alternating GM blocks at 4.6–4.7 ppm. In addition, the H-5 proton of guluronic acid in homopolymeric G blocks appeared at 4.4–4.5 ppm [58]. The three signals observed in this anomeric region correspond to the integrated areas A_1_, A_2_, and A_3_, which were quantified for each sample and are reported in Table 8, enabling the comparison of the relative abundance of anomeric environments and the overall structural purity among the different alginate sources.

The M/G ratio, each monomer (FG or FM), the diad sequences (FGG, FMM, FMG, and FGM), and triad sequences (FMGM, FMGG, and FGGM) were calculated using the Equations (5)–(10) [56,57]; these fractions are calculated using the peak areas shown in Table 8. For a more complete description of their sequence, it may be illustrative to use parameter ƞ, as defined by Equation (7).

For G units:

FG = AI/(AII + AIII)(1)

For M units:

FM = 1 − FG(2)

GG Fraction:

FGG = AIII/(AII + AIII)(3)

GM Fraction:

FGM = FMG = FG − FGG(4)

MM Fraction:

FMM = FM − FMG(5)

M/G = (1 − FG)/FG(6)

*n*
= FMG/(FM × FG) (7)

FMGM = FMG × FM(8)

FMGG = FMG × FG(9)

FGGM = FMG × FM(10)

According to Grasdalen and coworkers, ƞ values < 1 represent the abundance of homopolymeric blocks, meaning there are long sequences of either M or G units. This implies that the polymer chain contains significant stretches of either M-M-M (mannuronate block) or G-G-G (guluronate block) sequences, with minimal mixing of M and G units. In this case, all samples showed a value <1, representing the homogeneity of G blocks. Both extracted and commercial alginates exhibited low M/G ratios (<1) and high G fractions, as seen in Table 9.

Although there were similarities in the fractions of all the alginates, *S. polyschides* SA demonstrated a higher M content. However, the commercial variant displayed higher MM blocks, which are associated with hindering gel formation. The integral of a peak is proportional to the number of protons (hydrogen atoms) contributing to that peak [59]. The molecular weight and degree of polymerisation of the alginate samples can affect the NMR signal. The broader peaks in the commercial sample may be due to a higher degree of polymerisation or a higher molecular weight, resulting in stronger NMR signals, as a larger number of repeating units contribute to the signal [26,27].

The M/G ratio in alginate varies with the algae species, season, and origin, thus influencing gel properties. High M content yields soft, elastic gels, while high G content produces stronger, more rigid structures [60,61]. Low-molecular-weight alginate with high G content forms stable particles ideal for encapsulation [62]. High M content may stimulate immune responses [63], while high G content allows for better diffusion and crosslinking. In this case, the low viscosity observed may be due to the low M block content and higher G fractions [55].

### 2.3. Comparative Performance of the Extraction Protocol: Yield, Quality, and Operational Advantages

As already mentioned, the extraction of alginate from brown algae is highly dependent on the species, processing conditions, and chemical treatments employed. When comparing the protocol optimised in this study with those previously reported in the literature for both *S. muticum* and *S. polyschides*, clear advantages emerge in terms of the efficiency, simplicity, and product characterisation, as shown in Table 10.

For *S. muticum*, the proposed method yielded 23.2% alginate using only two main steps: acid pretreatment with 0.2 M HCl for 24 h, followed by alkaline extraction with 2% Na_2_CO_3_ for 2 h 30 min at 40 °C. This yield surpasses that of González-López et al. [64] (5–10.09%) and Mazumder et al. [65] (13.6 ± 0.1%) and is nearly equivalent to the highest reported (25.62%) by El Atouani et al. [21], which, however, involves a 72 h long, multi-reagent process, including formaldehyde and prolonged soaking steps. In contrast, the method developed in this study is more time-efficient, excluding toxic reagents such as formaldehyde, and is thus better aligned with environmentally conscious and industrially scalable practices. In addition to its high yield, the alginate extracted in this study exhibited desirable physicochemical properties. The molecular weight (4.91 × 10^4^ g/mol) and low viscosity suggest improved solubility and processability, which are essential for applications in biomedicine, microencapsulation, and cosmetics. The M/G ratio of 0.48, indicative of a higher mannuronic acid content, is associated with enhanced flexibility and gel-forming capabilities in softer matrices. Furthermore, the polyphenol content (0.0971 ± 0.0008 g GAE/100 g) was markedly lower than in other protocols (e.g., 1.92 ± 0.09 g GAE/100 g [64]), which is particularly relevant for applications that are sensitive to oxidation or requiring high alginate purity.

Similarly, for *S. polyschides*, the same two-step extraction protocol yielded 21% alginate, exceeding some values for traditional extraction (9–11.2%, [20]), and closely matching values reported in more complex and time-consuming approaches, including microwave-assisted extraction (23.8%, [20]) and formaldehyde-based extractions (20.19%, [19]). Importantly, the M/G ratio (0.58) and molecular weight (6.69 × 10^4^ g/mol) obtained in this study are well within the ideal range for gel-forming applications, indicating a structurally robust yet flexible material. The low polyphenol content (0.078 ± 0.0001 g GAE/100 g) again reinforces the quality of the extract, while the process simplicity (only two steps) positions this method as a strong candidate for scalable, cost-effective production.

Overall, the present study highlights that the optimised extraction protocol efficiently produces high-purity SA from both *S. polyschides* and *S. muticum* without the use of harsh chemicals and/or energy-intensive techniques. The combination of chemical, thermal, morphological, and spectroscopic analyses confirms that the extracted alginates retain their native structure, exhibit favourable M/G ratios, and possess homopolymeric G block sequences critical for gelation. While *S. muticum* presents slightly higher G content and stiffer gel potential, *S. polyschides* provides a more balanced profile with lower protein and polyphenol contamination, highlighting its suitability for biomedical and industrial applications. Overall, these results underscore the potential of these Portuguese macroalgae as sustainable and versatile sources of high-quality sodium alginate, bridging the gap between eco-friendly extraction processes and industrially relevant polymer properties.

**Table 10:** Comparison of different alginate extraction techniques from *S. muticum* and *S. polyschides* (the methodology and results explored in this research are in bold).

Algae	Extraction Technique	Maximum Yield of Extraction	Total Steps/ Additional Steps/Time	Characterisation				Reference
				Viscosity	MW (g mol^−1^)	M/G Ratio	Polyphenol Content	
*Sargassum muticum*	► Formaldehyde pretreatment + HCl + Na_2_CO_3_ extraction ^1^	14%	3/Formaldehyde (24 h)/72 h	n.a.	n.a.	1.08	0.236 ± 0.03 mg mL^−1^	[65]
	► Formalin hydration + HCl + Na_2_CO_3_ extraction ^2^	26%	3/Formaldehyde (24 h)/72 h	n.a.	n.a.	1.04	n.a.	[21]
	► Formaldehyde + H_2_SO_4_ + Na_2_CO_3_ extraction ^3^	5–10%	3/Formaldehyde/Sulphuric acid/ n.a.	n.a.	n.a.	n.a.	1.92 ± 0.09 (g GAE 100 g^−1^)	[64]
	**► HCl (24 h) + Na_2_CO_3_ (2.5 h) extraction ^4^**	**23%**	**2/27 h**	**Low**	**4.9 × 10^4^**	**0.48**	**0.10 ± 0.01** **(g GAE 100 g^−1^)**	**This study**
*Saccorhiza polyschides*	► HCl pretreatment + Na_2_CO_3_ extraction ^5^	23%	4/2× pretreatment HCl; 10% Alkali step/n.a.	n.a.	n.a.	n.a.	n.a.	[65]
	► Microwave-assisted extraction + CaCl_2_ precipitation ^6^	9–11%	3/Microwave-assisted extraction; 10% Alkali step/n.a.	n.a.	n.a.	<1	n.a.	[62]
	► Formalin + HCl + Na_2_CO_3_ extraction ^7^	23%	3/2% formalin/72 h	n.a.	0.73	0.81	n.a.	[17]
	**► HCl (24 h) + Na_2_CO_3_ (2.5 h) extraction ^8^**	**21%**	**2/27 h**	**Low**	**6.7 × 10^4^**	**0.58**	**0.08 ± 0.01** **(g GAE 100 g^−1^)**	**This study**

^1^. A 0.2% formaldehyde treatment (24 h and 16 °C) → washing with water → 0.2 M HCl treatment → washing → Na_2_CO_3_ extraction → centrifugation → precipitation of alginate with aqueous ethanol. ^2^. Hydration in 2% formalin for 24 h → washing → soaking in 0.2 N HCl for 24 h → extraction with 2% Na_2_CO_3_ for 24 h → filtration through cheesecloth → centrifugation of filtrate. ^3^. Soaking in 1% formaldehyde → extraction with 0.2 N H_2_SO_4_ → extraction with 1% Na_2_CO_3_ → intermediate washings with distilled water → precipitation with 95% ethanol → washing with absolute ethanol and acetone → drying at 40 °C for 24 h. ^4^. A 0.2 M HCl treatment (24 h, 40 °C, and 180 rpm) → washing → extraction with 2% Na_2_CO_3_ (2.5 h and 40 °C). ^5^. Conventional acid pretreatment: 1 M HCl to pH 4 (twice) → overnight stirring → addition of 10% Na_2_CO_3_ for extraction. ^6^. Microwave-assisted extraction (100 °C and 20 min) → precipitation of alginate with 10% CaCl_2_ → conversion of calcium alginate to alginic acid (1 M HCl) → washing with ethanol/water → pH adjustment to 8 with 10% Na_2_CO_3_ to obtain sodium alginate. ^7^. Soaking in 2% formalin for 24 h → washing → soaking in 0.2 N HCl for 24 h → extraction with 2% Na_2_CO_3_ for 24 h. ^8^. A 0.2 M HCl treatment (24 h, 40 °C, and 180 rpm) → washing → extraction with 2% Na_2_CO_3_ (2.5 h and 40 °C).

## 3. Materials and Methods

### 3.1. Materials

Sodium alginate (SA) was extracted from a brown seaweed collected at Praia dos Barcos, Baleal, Portugal; hydrochloric acid (HCl, ≥37%) from Honeywel (Wien, Austria) and anhydrous sodium carbonate (Na_2_CO_3_, ≥99.5%) from Sigma-Aldrich (St. Louis, MO, USA) were used for the sodium alginate extraction; methanol (CH_3_OH > 99.9%) and acetone (C_3_H_6_0) from Sharlau (Barcelona, Spain) were used to purify the alginate. Sodium alginate (C_6_H_7_NaO_6_,) from Sigma-Aldrich was used for comparison with extracted alginate. Folin–Ciocalteu reagent from VWR (Radnor, PA, USA) and gallic acid (C_7_H_6_O_5_, ≥98.5%) from Sigma-Aldrich (Darmstadt, Germany) were used in alginate characterisation tests.

### 3.2. Methods

#### 3.2.1. Sampling and Biomass Treatment

Specimens of the specie *S. polyschides* were collected at Praia dos Barcos, Baleal, Portugal (39°22′36″ N 9°20′26″ W). The harvesting conditions were favourable for sampling, with a tide of 0.8 m (at 11.45 am on 19 September 2023) and 0.6 m (at 8.31 am on 28 September 2023), and a water temperature of 19 °C on both days. The specimens were collected using scissors and cut off, leaving the base intact so that they could regrow. *S. polyschides* was collected upstream on the beach (39°22′35″ N 9°20′24″ W). Around 3 Kg of algae was collected and washed on-site with salt water to remove accumulated debris and sand. The process was repeated further north on the same beach (39°22′36″ N 9°20′25″ W), where it was possible to collect *S. muticum*, an invasive species, but less abundant at the site when compared to *S. polyschides*. A sample of around 2.5 Kg was collected.

After collection, the algae were first washed with salt water (3×), followed by running water (3×), and finally with distilled water (3×). The biomass was reduced in size to ~10 cm fractions, weighed, and dried in an oven for 48 h until a constant weight was achieved at 60 °C. With the aid of a coffee grinder (SilverCrest, SKMS 180 A1, Lidl Stiftung & Co. KG, Neckarsulm, Germany), the dried algae were ground into powder, sieved through a 1 mm mesh sieve, weighed to calculate the dry weight (DW), and stored in closed plastic containers protected from light with aluminium foil and kept at room temperature to protect against humidity.

#### 3.2.2. Alginate Extraction

In this initial optimisation step, the acid pretreatment time and alkaline extraction temperature were selected as the main variables based on their well-documented influence on alginate solubilisation and structural preservation [66]. Other parameters, including acid and alkali concentration, were kept constant to maintain a simple and scalable protocol.

#### Acid Treatment Optimisation

For the alginate extraction, improvements in acid treatment (time) and alkaline extraction (temperature) were performed separately.

Briefly, for the acid treatment, 5 g of dry biomass was added to 100 mL of 0.2 M HCl in a 250 mL Erlenmeyer flask, covered with aluminium foil, and placed at 40 °C with stirring at 180 rpm. The tests were carried out in quadruplicate (*n* = 4) and five treatment times were tested: 2, 12, 24, 32, and 40 h. After the predefined time, the extract was removed from the orbital and left to settle for 5 min. The liquid fraction was removed, and the biomass was washed with Milli-Q water, left to settle again, and the liquid fraction was discarded.

The alkaline extraction was then carried out by adding 100 mL of 2% (*w*/*v*) Na_2_CO_3_ and incubating for 2 h 30 min at 40 °C at 180 rpm. The resultant mixture was centrifuged at 6000× *g* for 30 min at 4 °C, and the supernatant was collected and precipitated with 96% ethanol (1:4 volume). After precipitation, a purification step was carried out to remove the phenolic compounds and pigments with successive washes in 70% ethanol, 96% ethanol, and 90% methanol and acetone (90%) with a 1:2 volume ratio. The precipitated alginate was air-dried, frozen at −80 °C, and freeze-dried for 48 h.

The wide time intervals used in the acid pretreatment step were selected to provide a broad screening of the process [66]. Although this approach may not capture a narrow optimum, the observed monotonic trend suggests a broad operational window rather than a sharp maximum. A finer time gradient could be explored in future studies to refine the optimal pretreatment duration.

#### 3.2.3. Optimisation of Alkaline Extraction Temperature

Based on the results above, an acid pretreatment time of 24 h was selected for further optimisation. The new tests were carried out with the same parameters for acid treatment and different alkaline treatment temperatures.

First, for the acid treatment, 5 g of biomass was added to 100 mL of 0.2 M HCl in a 250 mL flask, covered and placed at 40 °C, with stirring at 180 rpm. The tests were carried out in quadruplicate (*n* = 4); after 24 h, alkaline extraction was carried out with 100 mL 2% Na_2_CO_3_ for 2 h 30 min at 4 different temperatures (20 °C, 40 °C, 60 °C, and 80 °C). Temperature ranges were selected based on previous studies demonstrating the strong temperature dependence of alkaline extraction efficiency, which affects both polysaccharide solubilisation and the preservation of structural integrity [67]. Accordingly, a broad temperature range was evaluated to identify the optimal conditions for maximising extraction performance while maintaining alginate quality.

The collection, precipitation, and purification steps were repeated exactly as described in previous points. All samples were freeze-dried for 48 h; then, they were reduced to powder in a coffee grinder, weighed, and stored in 15 mL tubes.

#### 3.2.4. Statistical Analysis

Extraction yield was calculated according to Equation (11):

(11) $$Yield\;of\;extraction\;\left(\%\right)=\left(\frac{dried\;crude\;extraction\;weigth}{Initial\;weigth\;of\;algae\;sample}\right)\;\times \;100$$

Each experience was performed at least four times, and a statistical evaluation was performed by comparing the average values of the yield of extraction for each independent variable (algae, temperature, and time). Homogeneity tests were conducted to ensure homogeneity between the groups, allowing us to proceed with the statistical analysis. Data were analysed by a two-way ANOVA distribution using the Tukey Honestly Significant Difference (HSD) to determine statistically significant differences (*p* = 0.05) at the 95% significant level for each factor (algae, temperature, and time) in extraction yield. A multiple comparison procedure was applied to determine which means were significantly different from each other.

#### 3.2.5. Physicochemical Characterisation

##### Phenolic Compounds

The total polyphenols present in the extracted alginate can be estimated according to the methodology used by Singleton and Rossi, slightly modified [68]. A 1% alginate solution in water was prepared. Then, 1 mL of this solution was mixed with 5 mL of Folin–Ciocalteu reagent (FCR), diluted 1:10 (*v*/*v*) with distilled water. It was incubated at room temperature for 5 min, and then 4 mL of 6% (*w*/*v*) sodium carbonate was added and incubated, protected from light, for 90 min. The absorbance was measured at 765 nm. A standard curve was made with gallic acid at concentrations ranging from 0.1 to 5 mg/mL, and the total polyphenol concentration was calculated as gallic acid equivalents (GAEs) in mg (100 g^−1^) of dry alginate [65].

##### Protein Composition

The protein content was determined using a Pierce BCA Protein kit (Thermo Scientific, St. Louis, MO, USA), as described by the supplier. The alginate was dissolved in deionised water to obtain a final solution of 1% SA. To obtain the working reagent (WR), reagent A was added to reagent B in a 50:1 ratio, for a final volume according to that required for the number of samples desired. The calibration curve was made as described by the supplier. For the assays, 0.1 mL of each solution was added to 2 mL of WR and left to incubate for 2 h at room temperature, protected from light. After the 2 h had elapsed, the blank was made with Milli-Q water and the absorbance was measured at 652 nm, making dilutions if necessary. The protein concentration was calculated in BCA equivalents for the alginate samples.

##### Viscosity and Molecular Weight

Apparent viscosity was measured using a Brookfield HAAKEE viscotest 7 plus viscometer (Thermo eletron corporation, Dreieich, Germany). The measurements were performed in 250 mL of 0.4% (*w*/*v*) alginate solution at 20 °C after being subjected to 45 s of agitation and showing stable values. The spindle choice was made by trial and error; for a torque % between 10 and 100%, it was necessary to use the R2 spindle at 200 rpm. The result is expressed in mPas.s. and converted into g/mol. Three independent measurements were performed for each sample. The relative molecular weight was measured using Dynamic Light Scattering (DLS) (Zetasizer, Malvern, UK), starting with a 1% solution (10 mg/mL) of each alginate. Solutions were obtained at 5 different concentrations, ranging from 1 mg/mL to 10 mg/mL. The results were expressed in relative molecular weight (g/mol) and interactive diffusion parameters. The molecular weight, diffusion parameters, and coefficients are the average results of at least three independent measurements.

##### Fourier Transform Infra-Red Spectroscopy (FTIR)

The samples were incubated for 24 h at 37 °C before conducting the FTIR analysis so to eliminate accumulated humidity, as alginate is highly hygroscopic. Dried sodium alginate was placed in powdered form on the sensor for spectroscopic studies. FTIR spectra of sodium alginates (SAs) were recorded on an Alpha II compact FTIR (Burker, Bremen, Germany) with 64 scans. The results were processed using Opus vibrational spectroscopy software version 9.0.6), and the data were then processed. Samples were analysed and compared with commercially available SA.

##### Simultaneous Thermal Analysis (STA)

The thermal stability of the different alginates was simultaneously studied by differential scanning calorimetry (DSC) and thermogravimetric analysis (TGA). Results were obtained on a simultaneous thermal analyser (STA 6000, PerkinElmer, Bridgeport Ave Shelton, CT, USA) using 7 mg of the sample with a temperature range of 30–900 °C at a heating rate of 10 °C min^−1^ in a nitrogen atmosphere with a flow rate of 20 mL/min and an average pressure of 3 bar. A baseline was established under the same conditions; then, the samples corresponding to each tested extraction condition were run. The thermograms were separated in DSC, expressed in heat flow (mW) as a function of temperature (°C), and thermogravimetric analysis (TGA) expressed as mass loss (Δ mass) and its derivative (Δ mass %) as a function of temperature (°C). The respective peaks, on-set and maximum temperatures, and mass losses were calculated in Pyris™ software version 13.4.0.0064.

##### Scanning Electron Microscopy and Energy Dispersive X-Ray Spectroscopy

The morphology of SA was examined using scanning electron microscopy (SEM) (Vega 3, Tescan, Brno, Czech Republic). Before imaging, the samples were dried at 37 °C in an oven for a minimum of 24 h to remove any moisture. Subsequently, they were sputter-coated with gold using a sputter coater (SC7620, Korvus Technology, Buckinghamshire, UK). SEM was operated at an acceleration voltage of 15 kV, and images were captured at magnifications of 0.5 Kx, 1.0 Kx, and 5.0 Kx, with a maintained working distance of 10.98 mm. Additionally, for the morphology analysis, the samples underwent Energy Dispersive X-ray Spectroscopy (EDX) to quantify the elements present. This elemental analysis aimed to identify and quantify sodium (Na), carbon (C), and oxygen (O) in the samples.

##### Nuclear Magnetic Resonance (NMR) Spectroscopy

The M/G composition of CA and SA was determined by ^1^H NMR spectrometry according to the protocol proposed by Grasdalen et al., 1983 [62]. The ^1^H NMR spectra of commercial and extracted SA were successively recorded at 90 °C in D_2_O. The ^1^H NMR experiments were performed using a JOEL Instrument (Peabody, MA, USA) operating at 500 MHz. Previously, both samples were submitted to a mild acid hydrolysis of Triethylenetetramine-N, N, N′, N″, N‴, N‴-hexaacetic acid (TTHA). The ^1^H NMR spectra were collected using 128 scans and a relaxation time of 60 s.

## 4. Conclusions

The present study intended to optimise an extraction methodology for SA from the brown seaweeds *S. polyschides* and *S. muticum* and to complete the physicochemical characterisation. From the optimised protocol developed in this research work, an SA recovery higher than 20% (weight SA/weight dried alga) was achieved, representing an increase of almost 10% compared to similar methodologies used previously, and yielding a comparable result to some advanced techniques. A simple purification step decreased impurities, reduced toxic effects, and improved biocompatibility without producing relevant changes in the SA physicochemical characteristics. The protein content, total polyphenols, and STA helped to prove the purity of the extracted biomass, while SEM, EDX, and viscosity/molecular weight further characterised the extracted product by giving insights into its structure and composition, allowing us to infer the hydrogel-forming behaviour. The ^1^H-NMR spectroscopy revealed that the extracted alginates are richer in glucuronic acid (G) than mannuronic acid (M/G ratio < 1), thus providing strong hydrogels. FT-IR spectroscopy analysis showed an interesting similarity between the alginate spectra of the studied algae and that of the commercial alginate.

Nonetheless, the obtained results showed a very promising use of these algae for the extraction of SA. The simplicity of the protocol employed, the high yield of SA obtained, and the inexistence of aggressive solvents, surfactants, additional downstream steps, or conjugated technologies indicates the viability of using a waste biomass as a source of a product of interest with a high purity and promising characteristics. This reflects a more sustainable protocol both chemically and energetically.

## Figures and Tables

**Table 1:** *S. polyschides* ANOVA analysis of the extraction yield of the extraction considering the variables temperature (Temp) and algal species; * *p* > 0.05, and *** *p* > 0.001 for a significance level of 95%.

Source	Sum of Squares	Degree of Freedom	Mean Square	F-Value	*p*-Value	Significance
Model	368.455	9	40.929	12.261	<0.001	***
Intercept	10,758.057	1	10,758.057	3222.042	<0.001	***
Algae (×1)	18.25	1	18.250	5.466	0.026	*
Temp (×2)	334.517	4	83.629	25.047	<0.001	***
Algae × Temp	15.688	4	3.922	1.175	0.342	
Error	100.167	30	3.339			
Total	11,226.679	40				

**Table 2:** *S. muticum* ANOVA analysis of the extraction yield of the extraction considering the variables temperature (Temp) and algal species; * *p* > 0.05, and *** *p* > 0.001 for a significance level of 95%.

Source	Sum of Squares	Degree of Freedom	Mean Square	F-Value	*p*-Value	Significance
Model	219.279	7	31.326	15.436	<0.001	***
Intercept	11,610.224	1	11,610.224	5721.085	<0.001	***
Algae (×1)	33.143	1	33.143	16.332	<0.001	***
Temp (×2)	1676.367	3	55.789	27.491	<0.001	***
Algae × Temp	18.769	3	6.256	3.083	0.046	*
Error	48.705	24	2.029			
Total	11,878.208	32				

**Table 3:** Total polyphenols in (GAE) equivalent of different sodium alginates (R^2^ = 0.998).

Sample	[GAE] µg mL^−1^	mg (GAE) g^−1^ DW	Phenolic Compounds (%)
*S. polyschides*	77.6	0.08 ± 0.01	0.008
*S. muticum*	97.1	0.10 ± 0.01	0.010
Commercial	72.1	0.07 ± 0.01	0.007

**Table 4:** Protein content of different sodium alginates determined using the BCA method (R^2^ = 0.994).

Sample	[BCA] µg/mL	mg (BCA) g^−1^ DW	Protein (%)
*S. polyschides*	149.3	0.15 ± 0.01	0.016
*S. muticum*	664.3	0.69 ± 0.02	0.069
Commercial	71.3	0.08 ± 0.01	0.007

**Table 5:** Comparative values of relative Viscosity (mPa·s) and viscosity (cm^3^/g) obtained by Brookfield viscometer, and molecular weight (g/mol) and diffusion interaction parameter (mL/g) obtained by DLS.

Sample	Ƞ (mPa·s)	Ƞ (cm^3^ g^−1^)	Mw (Da)	R^2^ (Mw)	kD (mL g^−1^)
*S. polyschides*	33	3030	6.8 × 10^4^	0.99	−0.08
*S. muticum*	30	3000	4.9 × 10^4^	0.99	−0.08
Commercial	68	14,706	45 × 10^4^	1.00	−0.08

**Table 6:** Thermal events of SA samples measured by STA.

		T1			T2			T3	
Sample	T Onset °C	Peak °C	T Max °C	T Onset °C	Peak °C	T Max °C	T Onset °C	Peak °C	T Max °C
*S. polyschides*	30.0	67.5	121.0	204.2	236.2	267.4	787.6	863.1	892.6
*S. muticum*	32.3	70.1	123.1	202.6	236.8	264.5	768.8	854.3	889.6
Commercial	36.3	70.9	117.0	216.6	245.6	268.9	768.6	834.4	874.7

**Table 7:** EDX element quantification with normalised mass % and sodium–carbon (Na/C) ratio.

Sample	Wt (%)			
	C	O	Na	Na/C
*S. polyschides*	31.84	48.25	19.91	0.6255
*S. muticum*	43.89	44.51	13.15	0.2997
Commercial	40.56	48.57	10.87	0.2680

**Table 8:** Peak areas from the ^1^H NMR (5.3–4.3 ppm) analysis of commercial alginate and alginate extracted from *S. polyschides* and *S. muticum*.

SA Sample	A1—Integrated Area of Anomeric Peak 1	A2—Integrated Area of Anomeric Peak 2	A3—Integrated Area of Anomeric Peak 3
*S. polyschides*	1.28	1.07	0.95
*S. muticum*	1.1	0.81	0.82
Commercial	2.28	1.52	1.98

**Table 9:** Fractions, diad, and triad frequencies of SA quantified by ^1^H NMR.

	Fractions		Ratio	Diad			Triad			
Sample	FG	FM	M/G	FGG	FMM	FGM = FMG	FMGM	FMGG	FGGM	ƞ
*S. polyschides*	0.63	0.37	0.58	0.47	0.21	0.16	0.06	0.10	0.06	0.70
*S. muticum*	0.67	0.33	0.49	0.50	0.16	0.17	0.06	0.12	0.06	0.78
Commercial	0.65	0.35	0.54	0.57	0.27	0.09	0.03	0.056	0.03	0.38

## Data Availability

The raw data supporting the conclusions of this article will be made available by the authors on request.
